# Work absence in patients with asthma and/or COPD: a population-based study

**DOI:** 10.1038/s41533-021-00217-z

**Published:** 2021-02-16

**Authors:** Boudewijn J. H. Dierick, Bertine M. J. Flokstra-de Blok, Thys van der Molen, Núria Toledo-Pons, Miguel Román-Rodríguez, Borja G. Cosío, Joan B. Soriano, Janwillem W. H. Kocks, Job F. M. van Boven

**Affiliations:** 1 University of Groningen, University Medical Center Groningen, Groningen Institute for Asthma and COPD (GRIAC), Department of General Practice & Elderly Care Medicine, Groningen, The Netherlands; 2 General Practitioners Research Institute, Groningen, The Netherlands; 3 Department of Respiratory Medicine, Hospital Universitario Son Espases, and CIBERES, Madrid, Spain; 4 Instituto de Investigación Sanitaria de Baleares (IdISBa), Palma de Mallorca, Spain; 5 Primary Care Health Service, Servei de Salut de les Illes Balears, Palma de Mallorca, Spain; 6 Hospital Universitario de la Princesa, Universidad Autónoma de Madrid, Madrid, Spain

**Keywords:** Health care economics, Asthma, Chronic obstructive pulmonary disease, Public health

## Abstract

Chronic obstructive pulmonary disease (COPD) and asthma impact on work productivity, but their population-based burden and clinical predictors are understudied. In this observational, real-life study, work absence of 14,383 asthma and/or COPD patients present in the MAJORICA cohort (Spain) was compared with the general population. Using multivariable regression, we studied the association of work absence with demographic and clinical characteristics. Patients with asthma and/or COPD had more work absence than the general population (15.2% vs 8.9%, *p* < 0.0001). Patients with asthma had more often periods of work absence compared to patients with COPD (16.0% vs 12.8%, *p* < 0.0001). The number of days absent were, however, less in asthma than in COPD (median: 15 days [IQR: 5–51] vs 39 days [IQR: 13–134], *p* < 0.001). Patients with asthma–COPD overlap were in between (14.5% with absence; median: 27 days [IQR: 10–82]). Comorbid anxiety, allergic rhinitis, and sleep apnoea were independently associated with more work absence.

## Introduction

Back in 1969 was the first time it was formally acknowledged that asthma was responsible for a considerable number of days off work in men of working age (15–65 years) in the United Kingdom^1^. In more recent years, additional studies have further explored the impact of asthma on work absence and lost productivity, including the costs for the employer^2^, the cost for the patient^3^, caretaker^4^, healthcare worker^5^, parents of children with asthma^6^, asthma patients with allergic rhinitis^7^ and long-term disability^8^. Specific studies have been performed to look into the costs of occupational asthma^9,10^. Predictors for work absence, due to respiratory reasons, that have been identified so far include type of job, low forced vital capacity and occupational exposure to vapours, gas, dust or fumes^11–13^.

In the 2004 Global Asthma Insights and Reality Surveys, the international burden of absences from school in children and work loss in adults caused by asthma in the previous 12 months was investigated and varied between 17% in Europe and 27% in the Asian-Pacific region^14^. Work loss was uniform in adults, although there were some notable exceptions, possibly because of socio-cultural differences^14^. Furthermore, it was shown that in asthma patients with comorbidities, productivity loss was even 1.5 times higher than in asthma patients without comorbidity^15^. In a recent review^16^, it was highlighted that asthma patients have a considerable health-related productivity loss, up to 20.9% of the time an asthma patient worked.

Regarding chronic obstructive pulmonary disease (COPD)-related work absence, fewer studies have been performed. In the Confronting COPD survey, more than a third of COPD patients (35.7%) reported that their condition kept them off from working, limited their ability to work or had caused them working time loss in the past year^17^. This is likely to be an underestimation since most of these people were already retired; in subjects of 65 years, 45.3% reported work loss during the past year. Indeed, when also disability pensions and lost income is taken into account, the economic impact of COPD in patients of working age seems considerable^18–22^. In a recent study in Greece investigating the disease burden of COPD, almost one-fourth of the participants reported that they had missed work during the past 12 months due to their respiratory symptoms, with the mean number of days lost being ten^23^. Wacker et al.^24^ showed that the number of sick days reported by COPD patients increased by GOLD staging, from 3.7-fold (GOLD 1) to 5.6-fold (GOLD 4) compared with lung-healthy control subjects. One of the most comprehensive assessments was the international COPD uncovered survey that was performed in six countries^25^. This multinational study showed that 40% percent of the respondents with COPD, all in working age (45–67 years old), had been forced to stop working due to their COPD.

Although multiple studies studied work absence, most studies based their data on self-report surveys, and not on objective data collected through health records. Indeed, in most countries, health records do not contain accurate information to assess work absence and productivity loss. In addition, few studies included a complete regional population, including both asthma and COPD patients. Also, the comorbidities and clinical parameters that predict work absence in asthma and/or COPD are understudied. Lastly, no studies on work absence in asthma–COPD overlap (ACO)^26^ patients have been conducted.

Our main objective is to compare work absence between patients with asthma, COPD and ACO in a large, real-life respiratory population based on objective data. Secondly, this study aims to identify demographic and clinical parameters that predict work absence.

## Results

### Study population

**Fig. 1:**
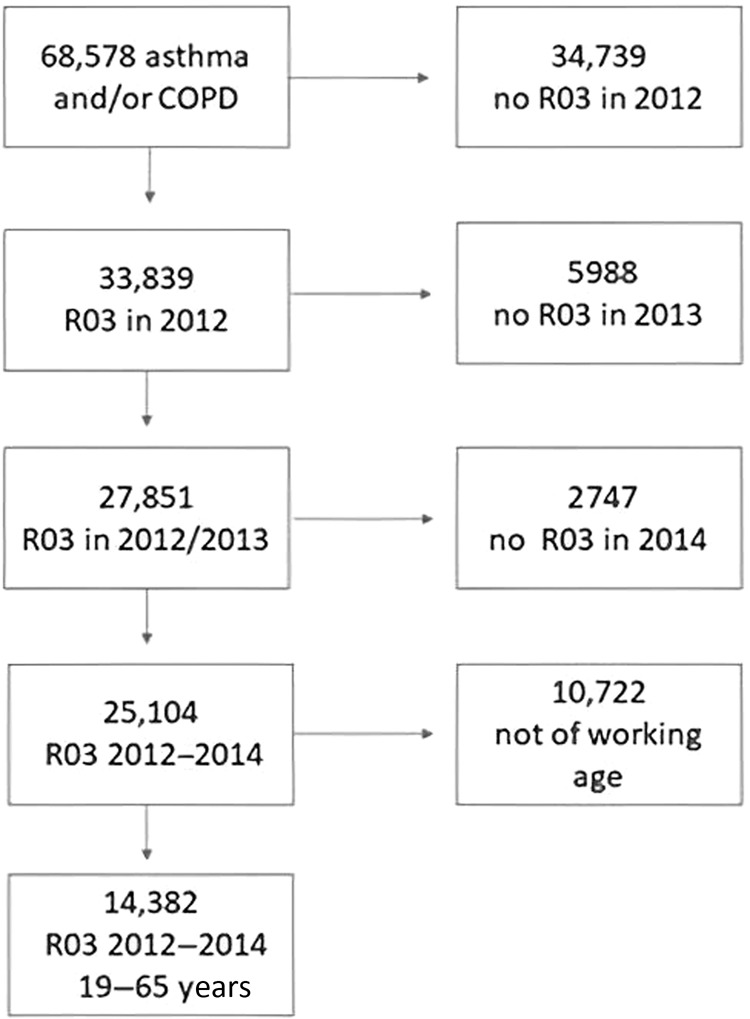
Flowchart 2012 MAJORICA database. Patients with asthma and/or COPD diagnosis on active ATC code R03 treatment and of workingage (19–65 years).

Overall in the MAJORICA cohort, there were 68,578 patients with asthma and/or COPD in 2012. To be sure we only selected patients with “active” disease and who were of working age, we only selected patients who were prescribed chronic respiratory treatment (ATC code R03) in the years 2012–2014 and were aged between 19 and 65 years old (Fig. 1). We analysed the work absence data and other data of the year 2012.

Table 1 provides the baseline characteristics of the asthma/COPD population. In total, there were 2843 (20%) patients with only a COPD diagnosis, 9988 (69%) patients with only an asthma diagnosis and 1551 (11%) patients with both asthma and COPD diagnoses from now onwards termed ACO. For the total asthma and/or COPD population, the most common comorbidities were anxiety (36%), allergic rhinitis (26%) and hypertension (25%).

**Table 1:** Baseline characteristics of the Balearic asthma and/or COPD population between 19 and 65 year-old (*n* = 14,382).

	Total respiratory population		Asthma alone		COPD alone		ACO	
	*N*	% or mean (SD)	*N*	% or mean (SD)	*N*	% or mean (SD)	*N*	% or mean (SD)
Demographics								
Age	14,382	46 (13.0)	9988	42 (12.3)	2843	58 (6.6)	1551	53 (9.7)
Male	6696	47	4017	40	1965	69	714	46
BMI	7509	29 (6.7)	4697	29 (6.7)	1806	30 (6.6)	1008	31 (7.0)
FEV1 %predicted	4633	75 (21.4)	2421	84 (18.1)	1452	63 (19.6)	760	68 (20.2)
Never smoker	5430	38	4687	47	308	11	435	28
Former smoker	2471	17	1229	13	876	31	366	24
Current smoker	4137	29	2124	21	1408	50	605	39
Unknown smoking status	2344	16	1948	20	251	9	145	9
Comorbidities								
Hypertension	3578	25	1700	17	1294	46	584	38
Heart failure	339	2	72	1	203	7	64	4
Ischaemic heart disease	591	4	173	2	333	12	85	5
Atrial fibrillation	637	4	283	3	257	9	97	6
Cor pulmonale	74	1	16	0.2	42	1	16	1
Stroke	324	2	115	1	167	6	42	3
Diabetes	1536	11	582	6	686	24	268	17
Depression	269	2	143	1	75	3	51	3
Anxiety	5120	36	3467	35	980	34	673	43
Osteoporosis	1272	9	748	7	304	11	220	14
HIV	30	0.2	9	0.1	16	1	5	0.3
Lung cancer	51	0.4	3	0.03	38	1	10	1
CKD	119	1	26	0.3	71	2	22	1
Allergic rhinitis	3737	26	3187	32	253	9	297	19
GERD	949	7	602	6	200	7	147	9
Sleep apnoea syndrome	750	5	300	3	321	11	129	8

*ACO* asthma COPD overlap, *BMI* Body Mass Index, *COPD* Chronic Obstructive Pulmonary Disease, *CVA* Cerebrovascular Accident, *FEV1* forced expiratory volume in 1 second, *GERD* Gastroesophageal reflux disease, *HIV* human immunodeficiency virus, *SD* standard deviation.

Table 1 also shows the baseline characteristics by respiratory diagnosis. Compared to asthma patients, COPD patients were more often males (69.1% vs 40.2%, *p* < 0.01), were older (57.9 years vs 42.0 years, *p* < 0.0001), had a worse FEV1% predicted 62.6% vs 83.9%, *p* < 0.0001) and a higher percentage of current- and ex-smokers (80.5% vs 33.6%, *p* < 0.01) For this normally distributed we applied the Student’s *T* test.

### Work absence

**Fig. 2:**
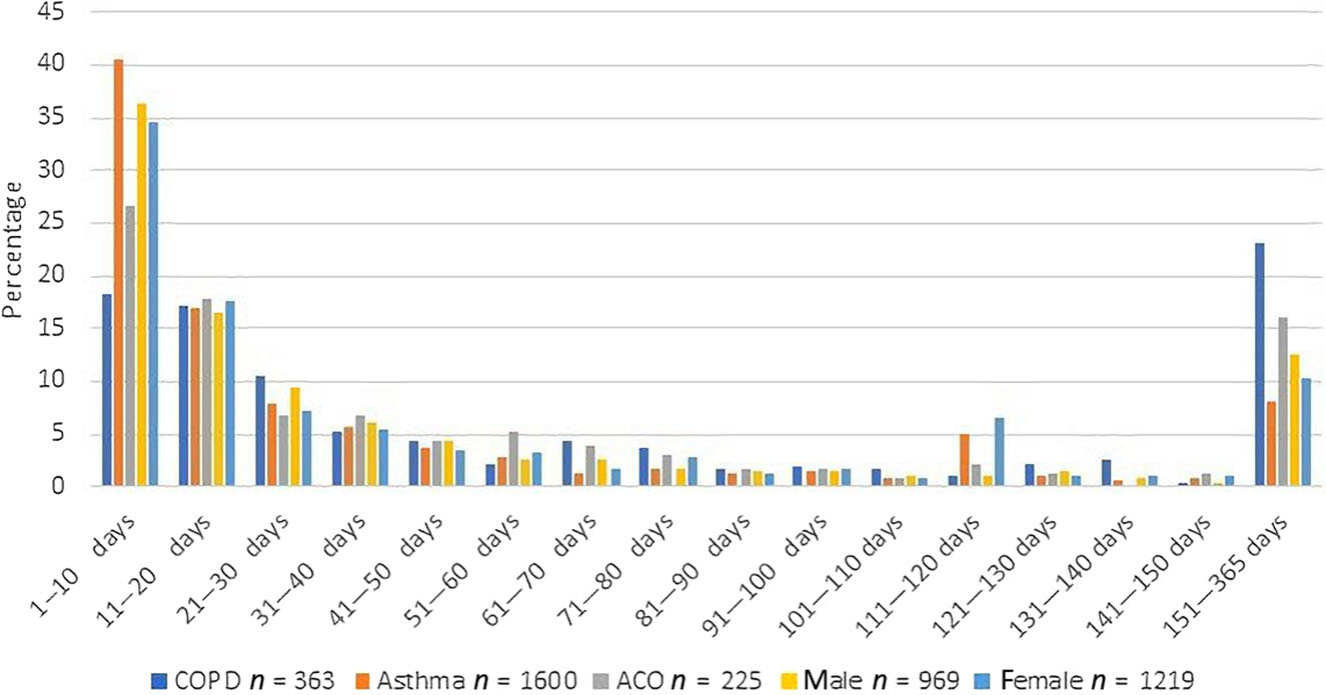
Distributions of days absent (%) from work in Balearic respiratory patients with any work absence (*N* = 2188).

The percentage with—and duration of—work absence in those with asthma and/or COPD of working age was compared with the Balearic general population of working age (Table 2). Note that work absence data was non-normally distributed (see difference in mean and median and Fig. 2) and therefore we used Mann–Whitney *U* tests to compare the median days of work absence. Patients with asthma and/or COPD had more work absence (15.2% vs 8.9%, *p* < 0.0001) than people of the Balearic general population and these work absence periods were longer (median: 18 [IQR: 5–53] vs 13 days [5–44], *p* < 0.001).

**Table 2:** Work absence in the Balearic asthma and/or COPD population and the Balearic general population aged 19–65 years in 2012.

	*n*	Work absence, *n* (%)^#^	Work absence days, mean (SD)	Work absence days, median (IQR)	*P*
General Balearic population					
Total	753,591	67,116 (8.9)	42.4 (72.8)	13 (5–44)	
Men	383,428	30,417 (7.9)	44.4 (75.7)	13 (5–45)	
Women	370,163	36,699 (9.9)	40.8 (70.3)	13 (5–42)	
Respiratory population					
Total	14,382	2188 (15.2)	57.5 (87.1)	18 (5–53)	<0.0001*
19–35 yr	3435	608 (17.7)	38.5 (61.5)	11 (5–41)	<0.0001*
36–50 yr	4674	776 (16.6)	49.5 (78.8)	16 (6–50)	0.001*
>50 yr	6273	804 (12.8)	79.7 (104.7)	32 (11–107)	0.001*
Men	6696	969 (14.5)	58.7 (93.1)	18 (5–48)	<0.0001*
Women	7686	1219 (15.9)	56.6 (82.1)	18 (5–57)	<0.0001*
Asthma					
Total	9988	1600 (16.0)	47.4 (75.5)	15 (5–51)	0.001*
19–35 yr	3310	580 (17.5)	38.5 (62.2)	11 (5–40)	<0.0001*
36–50 yr	3918	662 (17.3)	45.7 (73.1)	15 (5–49)	0.055
>50 yr	2760	358 (13.0)	64.9 (94.3)	24 (8–75)	0.395
Men	4017	608 (15.1)	36.9 (66.7)	12 (5–34)	0.022*
Women	5971	992 (16.6)	53.6 (79.8)	17 (6–73)	<0.0001*
COPD					
Total	2843	363 (12.8)	93.2 (112.7)	39 (13–134)	<0.0001*
19–35 yr	20	1 (5)	11 (0)	0	0.885
36–50 yr	357	45 (12.6)	97.4 (120.1)	43 (13–132)	<0.0001*
>50 yr	2466	317 (12.9)	92.6 (111.5)	39 (13–135)	<0.0001*
Men	1965	241 (12.3)	102.5 (119.4)	44 (14–157)	<0.0001*
Women	878	122 (13.9)	74.1 (95.8)	30 (11–89)	<0.0001*
ACO					
Total	1551	225 (14.5)	72.5 (99.1)	27 (10–82)	<0.0001*
19–35 yr	105	27 (25.5)	39.0 (42.3)	14 (5.5–82)	0.121
36–50 yr	399	69 (17.3)	54.5 (86.2)	17 (10–53)	0.023*
>50 yr	1047	129 (12.3)	89.0 (109.6)	41 (11–118)	0.008*
Men	714	120 (16.8)	81.6 (110.1)	33 (11–78)	<0.0001*
Women	837	105 (12.5)	62.0 (84.0)	22 (9–85)	0.001*

*ACO* asthma-COPD overlap, *COPD* Chronic Obstructive Pulmonary Disease, *SD* standard deviation, *IQR* Inter Quartile Range (*p* = median work absence days compared to general Balearic population) #percentage of individuals who had any work absence (1–364 days).

*p < 0.05, compared to general Balearicpopulation with work absence.

Patients with asthma had more often a period of work absence compared with patients with only the diagnosis of COPD (16.0% vs 12.8% (*p* < 0.0001), but these periods were much shorter in asthma patients than in COPD patients (median: 15 days [IQR: 5–51] vs 39 days [IQR: 13–134], *p* < 0.001). Patients with ACO are in between the aforementioned groups (14.5%; median: 27 days [IQR: 10–82]).

### Duration of work absence

Figure 2 shows the distribution of the duration of the cumulative periods of work absence in the respiratory population with any work absence (*N* = 2188). Except for COPD, most patients (27–41%) were absent between 1 and 10 days. Notable is the spike at 111–120 days in the female subgroup. This spike is mostly caused by the maternity leave of some women; in this interval in the total respiratory group, men and women, 83% of the work absence was caused by maternity leave. In Spain, the maternity leave is 110 days by law. A total of 86 patients (4%) were impaired to work for at least one year.

### Causes of work absence

**Fig. 3:**
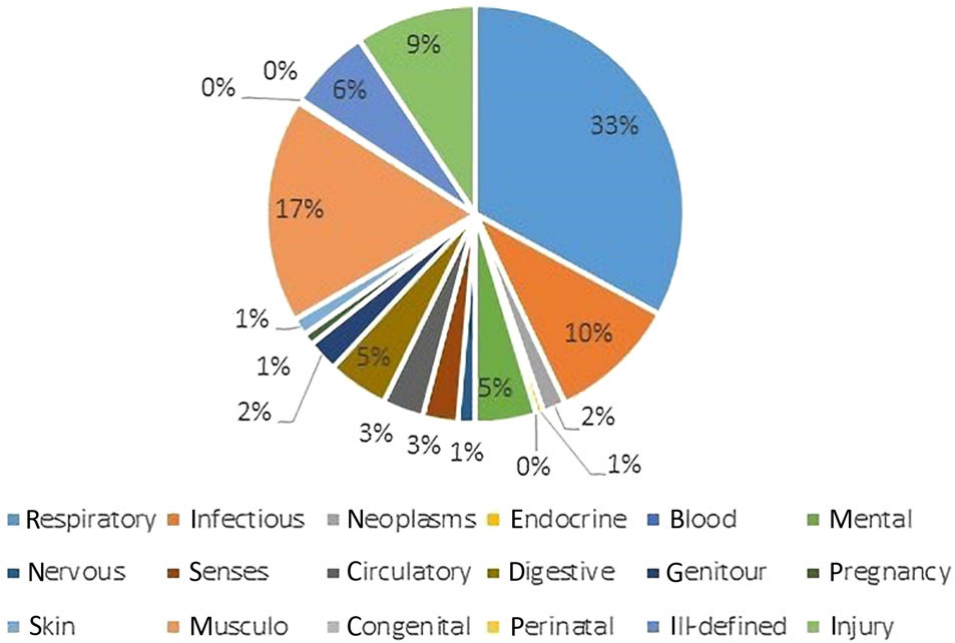
Diagnoses causing work absence in the respiratory population.

**Fig. 4:**
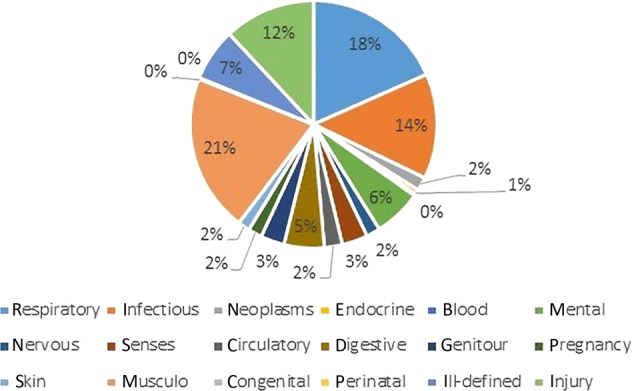
Diagnoses causing work absence in the general Balearic population.

Figures 3 and 4 show the causes of work absence by ICD-9 coding groups. In the Balearic general population, 18% of the periods of work absence were respiratory related. In patients with asthma and/or COPD, this was the case in 33% of the work absence periods (*p* < 0.0001). In addition, in both groups the musculoskeletal system was a major cause of work absence. Although anxiety is a very common comorbidity in the respiratory group (36%), only 5% of the work absence in the respiratory group is actually caused by psychiatric problems.

### Associations with work absence

Table 3 shows the influence of age, sex and comorbidities on work absence. Of note, age and having anxiety, sleep apnoea or allergic rhinitis showed an independent association with higher work absence in patients with asthma and/or COPD.

**Table 3:** Associations with work absence.

Variable	Univariate OR	95% CI		*P* value	Multivariate OR^#^	95% CI		*P* value
		lower	Upper			Lower	Upper	
Age 18–35	Ref	Ref	Ref		Ref	Ref	Ref	
Age 36–50	0.949	0.842	1.070	0.394	0.941	0.833	1.063	0.328
Age >50	0.710	0.632	0.798	0.000*	0.738	0.640	0.850	0.000*
Male (ref = female)	0.898	0.819	0.984	0.021*	0.968	0.878	1.067	0.511
Asthma	Ref	Ref	Ref		Ref	Ref	Ref	
COPD (ref = asthma)	0.767	0.679	0.867	0.000*	0.941	0.813	1.067	0.414
ACO (ref = asthma)	0.890	0.765	1.035	0.129	0.988	0.842	1.159	0.882
Hypertension	0.856	0.768	0.954	0.005*	0.991	0.876	1.121	0.885
Heart failure	0.784	0.566	1.087	0.144	0.934	0.664	1.314	0.695
Cardiomyopathia	0.778	0.606	0.998	0.048*	0.926	0.713	1.203	0.564
Atrial fibrillation	1.001	0.803	1.249	0.992				
Cor pulmonale	1.420	0.804	2.507	0.227	1.582	0.890	2.814	0.118
CVA	0.781	0.559	1.091	0.147	0.920	0.654	1.294	0.633
Diabetes	0.838	0.718	0.979	0.026*	0.974	0.822	1.154	0.761
Depression	0.944	0.670	1.330	0.742				
Anxiety	1.359	1.239	1.492	0.000*	1.369	1.244	1.506	0.000*
Osteoporosis	0.867	0.733	1.024	0.094	0.901	0.758	1.070	0.234
HIV	1.115	0.426	2.915	0.825				
Lung carcinoma	1.037	0.487	2.209	0.925				
CKD	0.454	0.230	0.897	0.023*	0.557	0.279	1.113	0.097
Allergic rhinitis	1.221	1.104	1.350	0.000*	1.135	1.023	1.259	0.017*
GERD	0.953	0.792	1.148	0.615				
Sleep apnoea	1.179	0.970	1.433	0.097	1.337	1.091	1.638	0.005*

*COPD* chronic obstructive pulmonary disease, *ACO* Asthma COPD overlap, *C.I* confidence interval, *CVA* cerebrovascular accident, *CKD* chronic kidney disease, *GERD* gastroesophagal reflux disease. *p < 0.05 uni(multi)variate regression analysis, *OR* odds ratio, *Resp.population* patients with asthma and/or COPD; # only those variables with *p* < 0.25 in the univariate analyses were included in the multivariable analyses.

## Discussion

This population-based study showed that patients with asthma and/or COPD have significantly more work absence than people in the general population. Generally, work absence was more frequent in the asthma population, but of longer duration in the COPD population. Within the asthma/COPD population, 33% of the work absence was caused by respiratory problems (vs 18% in the general population) and the other two-thirds by other causes such as musculoskeletal and infectious causes. Anxiety, sleep apnoea and allergic rhinitis showed an independent association with work absence in patients with asthma and/or COPD.

Recently, a number of studies have shown an association between COPD and/or asthma with work productivity and work absence^27–30^. Most studies had their own strengths, but none of them studied the causes of the work absence using a population-based approach and took into account objectively registered comorbidity data and work absence data.

Ding et al.^27^ studied the effect of asthma and/or COPD on work productivity. Over 2100 patients with COPD consulting for routine care in Europe, China and the USA were studied. Data for work productivity were derived from self-reported questionnaires. They studied the relation between increased COPD symptom burden and absenteeism and presentism. Over the 7 days prior to the study period, a significant association was observed between increasing absenteeism and an increasing COPD Assessment Test (CAT) score, yet total annual absence in days was not reported.

A recent systematic review by Rai et al.^28^ studied COPD and work-related outcomes. They selected 44 studies from 1937 to 2017. Thirty-five of these studies studied absenteeism, 13 studies compared absenteeism in people with or without COPD. Although these studies have been conducted in a variety of settings and populations, they showed higher absenteeism in people with COPD. In contrast with our study, the majority of these 44 studies did not use a population-based approach, used self-report data only and did not give an insight in the causes of the absenteeism.

Also, De Sousa Sena et al.^29^ studied work productivity, absenteeism and presentism in patients with COPD compared to non-COPD control subjects. The study provided a comprehensive overview of work productivity loss (absenteeism and/or presentism) among a representative population-based sample of individuals with mild to moderate COPD. They showed that work productivity loss was significantly higher in COPD subjects with a high symptom burden (CAT > = 10) compared to COPD subjects with a low symptom burden (CAT < 10) and control subjects, but did not focus on other causes of absenteeism.

In 2018, Lisspers et al.^30^ published a real-world retrospective cohort study of patients managed in primary care in Sweden and an age- and sex-matched reference population. They studied a total of 17,479 patients with COPD. In line with our study, this study showed a significant higher mean number of sick days in COPD patients (44.3 days) compared with the reference population (30.4 days). The study did not assess the causes of the work absence. Although this study analysed the prevalence of comorbidities, it did not study the relation between work absence and comorbidities.

A recent study analysed the work absence data of 348 patients with COPD in Birmingham, UK^31^, by means of a cross-sectional analysis of baseline data from a subsample (those in paid employment) of the Birmingham COPD Cohort Study. Subjects who self-reported having taken any time off work during the last 12 months were classified as exhibiting absenteeism. The cause of the absenteeism (respiratory, other health problems or other) and the duration of the absenteeism were also noted. They showed relatively high rates of absenteeism in patients with COPD and showed a relation between absenteeism and age. Also, this study showed that high all-cause absenteeism was more common in those with ≥1 comorbidity. In contrast with our study, this study did not specify which comorbidities were associated with more work absence.

Ervasti et al.^32^ studied all full-time employees working for 10 municipalities and 6 hospital districts in Finland. All subjects were of working age and had at least one depression-related absence during the observation period 2005 to 2011 (*n* = 10,875). They showed that patients with depression and asthma had a lower likelihood of returning to work than patients with depression and no comorbidity. Our study confirms that anxiety has indeed a negative influence on work absence in patients with asthma and/or COPD. As for the other comorbidities that were associated with higher absenteeism, sleep apnoea, allergic rhinitis and anxiety are highly symptomatic, affecting physical and mental work ability and often more difficult to treat as compared to other serious, but often less symptomatic comorbidities such as hypertension, chronic kidney disease or osteoporosis. Furthermore, sleep apnoea, allergic rhinitis and anxiety are symptomatic from the start and therefore may have a direct negative effect on productivity, whereas other diseases often start to be symptomatic later in life.

The aforementioned studies show that having asthma and/or COPD has a negative effect on work absence and on productivity. Our study further supports these conclusions. Not only self-reported symptoms, but also the pathophysiological nature of the diseases makes it logical that patients with asthma and/or COPD have more work absence. We think that showing the real-world effects of asthma and/or COPD on the work absence of these patients can be an important addition to the knowledge gained in earlier studies.

A major strength of this study is the fact that we had access to an entire regional population. All outcomes were objectively measured and structured, creating a large and unbiased sample. In Spain, people are obliged to visit their general practitioner (GP) before they can have sick leave. The GP not only registers the sick leave, but also the cause of the sick leave. In other studies, work absence was registered in different ways. Yet, often the cause of work absence was not registered at all or it was registered by surveys.

Some potential limitations of our study are worth discussing. Due to the use of retrospective data, we could not be certain which criteria were used to make the diagnosis. In practice, in the Balearics, as in numerous other countries, there are different paths to any diagnosis. Some are diagnosed by a family-, pulmonary- or other physicians, and have had all the tests needed, some are diagnosed only using spirometry and some are diagnosed a long time before. Unfortunately, we could not distinguish between these groups (being it asthma, COPD or ACO). However, given we only included patients who were on active respiratory treatment, effects of under and over-diagnoses are expected to be acceptable.

In our regression analyses, we did not include FEV_1_ because of missing data, but especially because FEV_1_ only has a poor relationship with clinical symptoms^33^. The fact that patients in Spain have to see their GP before they can officially call in sick may have had an effect on the incidence of work absence, people may sometimes just take some days of instead of calling in sick. The median and mean number of days with work absence may also be influenced by this. Yet, considering the fact that this has the same effect on the study group as on the total population, we do not think this influenced our conclusion significantly.

Unfortunately, we had no insight in what kind of work people did, for example, we could not distinguish between white- and blue collar workers. Also, we did not know which people were incapacitated for work and who were unemployed. The same holds true for information regarding income, ethnicity or educational background.

Our results showed that COPD and asthma influence the lives of people of working age considerably. It seems important to consider the effects of these diseases on people of working age and to start treating patients with asthma and/or COPD as soon as possible, in particular those with comorbid allergic rhinitis, anxiety and/or sleep apnoea. Preventive support seems to be of utmost importance. Further studies will have to be performed to ascertain the costs of increased work absence in patients with asthma and/or COPD. When these costs are clearly identified, policymakers will be more prone to facilitate more and better asthma and COPD research and to support the implementation of (preventive) care of these patients. As such, policymakers can take into account the full socioeconomic benefits of healthcare instead of only looking at the costs of healthcare.

Patients with asthma and/or COPD have significantly more work absence compared to the general population. Work absence is more frequent in asthma but is of longer duration in patients with COPD. Anxiety, allergic rhinitis and sleep apnoea could partially drive these effects.

## Methods

### Study design

This was an observational, cross-sectional, population-based study that characterizes the asthma and/or COPD population with work absence assessing the numbers of days absent from work in the Balearic Islands, Spain. The reporting of this manuscript followed the STROBE checklist for cross-sectional studies (Supplementary Information).

### Data source

Data were extracted from the MAJOrca Real-world Investigation in COPD and Asthma cohort (MAJORICA). MAJORICA contains data from the primary care system, the hospital system and the electronic prescription system in the Balearic Islands, Spain^34^. The database covers almost all clinical characteristics and healthcare utilization of the residents of the Balearics (±1.1 million subjects). More specifically, MAJORICA contains data from all patients ≥18 years with a primary care diagnosis of asthma and/or COPD in 2012, irrespective of health insurance, with at least 2 years follow-up available. From almost 70,000 respiratory patients, data were available on demographics, clinical data, diagnostic tests, health resource use, pharmacy dispense data, work absence data and patient-reported outcomes for the period 2011–2015. MAJORICA has been used in previous observational studies^35,36^.

### Study population

All MAJORICA patients with a physician-registered diagnosis of asthma and/or COPD (ICD-9 code 493 and/or ICD-9 code 491-492) who had a prescription of a respiratory treatment (defined by using at least one prescription with Anatomical Therapeutic Chemical code R03 annually) between the years 2012 and 2014, and were of working were selected. Only people ≥19 were included to be sure that all participants were at least one full year of working age. Patients who had both the diagnosis asthma (ICD-9 code 493) and the diagnosis COPD (ICD-9 code 491-492) were considered as ACO patients. The respiratory patients were compared with the total general Balearic population, also aged between 19 and 65 years old.

### Registration of work absence

In the Balearic Islands, the GP plays a crucial role in keeping a record of a patient’s work absence. When a patient is unable to work, he/she is obliged to consult the GP. The GP registers when the work absence starts, registers the primary diagnosis that causes the work absence (by ICD-9 code) and registers when the work absence ends. Patients cannot restart working before being registered as recovered by their GP. A period of absence lasts up to a maximum of 364 days. After 364 days, a patient is considered to be disabled.

### Outcomes

The primary outcome of this study was to investigate the difference in the percentage of people with any work absence between the asthma/COPD population and the Balearic general population. Secondary outcomes were: (1) the duration of work absence; (2) the causes of work absence; and (3) the predictors for work absence within the asthma/COPD population.

### Statistical analysis

To compare baseline characteristics of the study groups, we used Student’s *T*-tests (for normally distributed continuous variables) and Chi-square test and Fisher exact tests for categorical variables. To compare independent non-normally distributed data, we used the Mann–Whitney *U* test. To study the influence of demographics and comorbidities on any work absence, univariate logistic regression analyses were performed followed by multivariate regression analyses. In the univariate analyses we considered a *p* value lower than 0.25 for possible statistical significance^37^. In the multivariate analyses, a *p* value lower than 0.05 was considered for statistical significance.

### Ethics approval

The Balearic Primary Care Research Committee assessed and approved the study’s protocol. Because of the retrospective design and use of anonymized data, this study was exempted from ethics approval.

### Reporting summary

Further information on research design is available in the Nature Research Reporting Summary linked to this article.

## Supplementary information

Supplementary Information

Reporting Summary

## Data Availability

The datasets generated during and/or analysed during the current study are available from the corresponding author on reasonable request.

## Supplementary information

**Supplementary information** The online version contains supplementary material available at 10.1038/s41533-021-00217-z.

## Code availability

The codes generated during and/or analysed during the current study are available from the corresponding author on reasonable request.
